# Genomic research in Zambia: confronting the ethics, policy and regulatory frontiers in the 21st Century

**DOI:** 10.1186/s12961-015-0053-4

**Published:** 2015-10-29

**Authors:** Pascalina Chanda-Kapata, Nathan Kapata, Albertina Ngomah Moraes, Gershom Chongwe, James Munthali

**Affiliations:** Directorate of Disease Surveillance Control and Research, Ministry of Health, Lusaka, Zambia; National TB/Leprosy Programme, Ministry of Community Development, Mother and Child Health, Lusaka, Zambia; Department of Public Health, University of Zambia, Lusaka, Zambia; School of Medicine, University of Zambia, Lusaka, Zambia

**Keywords:** Ethics, Genomics, Legal, Policy, Recommendations, Regulations, Research, Zambia

## Abstract

Genomic research has the potential to increase knowledge in health sciences, but the process has to ensure the safety, integrity and well-being of research participants. A legal framework for the conduct of health research in Zambia is available. However, the ethical, policy and regulatory framework to operationalise genomic research requires a paradigm shift. This paper outlines the current legal and policy framework as well as the ethics environment, and suggests recommendations for Zambia to fully benefit from the opportunity that genomic research presents. This will entail creating national research interest, improving knowledge levels, and building community trust among researchers, policymakers, donors, regulators and, most importantly, patients and research participants. A real balancing act of the risk and benefits will need to be objectively undertaken.

## Background

Genomics is defined as the study of genes and their functions, and related techniques [1, 2]. The human genome project – an international, collaborative research program – has provided a complete map and understanding of the human genome [3, 4]. With the analysis of the human genome comes the opportunity to study biomedical research at a more finite level than has been previously possible. Advancements in technology have made it possible to rapidly analyse genetic information and elucidate its research and clinical relevance. This has given way to predictions that vaccines, drugs and other interventions will eventually be tailored according to an individual’s genetic make-up [5, 6]. The potential of genomics research to improve health outcomes of populations cannot be underestimated, and it is therefore important to determine the best way to progress quickly [6, 7]. However, there remains scepticism regarding the added value of genomics in disease prevention, with the notion that, rather, reinforcing population-based approaches to prevention, especially for diseases with known environmental causes, is more beneficial [8]. Khoury et al. [9] argue that applied genomic research is as important for conditions with environmental causes as for those without known environmental determinants.

Genome-wide association studies are among the genomic tools being used to identify the genetic contributors of common disorders such as diabetes, cardiovascular disease, and prostate cancer [10]. To date, only seven such studies have been documented to have been conducted exclusively on African participants [11, 12], and a few other studies have included some African participants [13–16].

The potential health benefits of genomic research are not always immediately tangible [17]. Genetic counselling and testing for hereditary syndromes are some of the few evidence-based applications that have become part of routine healthcare [18–20]. Therefore, a comprehensive genomic research agenda must be adopted in order to aid the translation of genomic research findings into healthcare in a way that maximizes health benefits and minimizes harm to individuals and populations.

Khoury et al. [21], in their review paper, provide a four-phase continuum framework for the translation of genomic research into healthcare and prevention that revolves around the development of evidence-based guidelines. The Clinical and Translational Research Institute in San Diego has implemented a model adapted from this framework [22]. However, Pawson et al. [23] suggest an alternative approach; their method focuses on context and external validity, and seeks to answer the questions: which intervention, for which problem, which set of patients is the intervention most effective for, and what outcome does it produce? Genomic research is both an opportunity and a challenge for all stakeholders, ranging from legislators, policymakers, researchers, ethics committees, and research participants, as there is an urgent need to balance the obligations to respect and protect research participants with social interest in advancing beneficial research [24].

In Zambia, genomic research, although rare, is an evolving science and the studies conducted so far have mainly focused on the key genetic determinants of the responses to HIV infection in a cohort of discordant couples [25] and on the sequencing of *Bacillus anthracis* outbreak strain CZC5 during the Chama district Anthrax outbreak of 2011 [26]; both these studies being important for the advancement of health service provision, health promotion, research and, subsequently, disease elimination. However, the cardinal issue is to ensure there is adequate regulation, an enabling environment and consensus on what constitutes genomics and informatics as argued by Lyon and Segal [6]. In this article, we discuss genomic research in Zambia, interrogating the issues pertaining to the ethics, policies and regulatory frontiers, and discussing the challenges being faced in this century, with recommendations for improving the status quo.

## Ethics environment

Genomic research is fraught with unique ethical challenges which will need the attention of regulators. These issues may not be adequately dealt with by current local and international guidelines and legal frameworks, and concern, in particular, current guidelines related to informed consent procedures, withdrawal from a research study, disclosure of research results which may result in adverse consequences, release of data to the public resulting in loss of privacy, and possible commercialisation of results [27]. Foremost among the ethical challenges to genomic research in Africa are the sensitivities and questions raised by communities based on their previous experiences and regional/cultural beliefs and practices [28].

All Research Ethics Committees (RECs) or Institutional Review Boards (IRBs) are supposed to be registered with the National Health Research Council, in accordance with the Health Research Act No. 2. 2013 of the Laws of Zambia, and should adhere to these principles.

According to the East, Central and Southern Africa Health Community assessment [29], a number of weaknesses/non-conformities in the Zambian ethics system were identified, including the lack of an on-going quality improvement system, no full-time employees assigned to the IRB/REC, no specific application form available for submission of protocols for approval, and protocols were not reviewed by regional or international bodies. There was no policy on the review process. In addition, the qualifications of the author to conduct the research were not taken into consideration. The interval of continued review for risky studies was not determined, neither was the input of a Community Advisory Boards or the availability of a product or utility to the community upon completion of the study taken into consideration. Community Advisory Boards are often associated with the review of clinical research ethics with regards the human subjects of research and serve as an aspect of community-based participatory research [30, 31]. In order for genomic research in Zambia to be successful, issues such as community engagement, broad consent, and the implications of sharing genetic information need to be debated and the potential risks of stigmatisation and harm need to be considered carefully [32]. Ultimately, these ethical challenges need to be weighed against strategies and approaches for making use of genomic research to address the high burden of disease in Zambia.

According to the Health Research Act No. 2 of 2013 of the Laws of Zambia, consent needs to be specific; therefore, broad and ‘open’ or ‘unrestricted’ consent is not possible in Zambia, and neither is long-term storage of samples. To what extent will it be necessary, for example, to modify current guidelines on informed consent to provide for research in future, unknown studies – which is a hallmark of bio-banking and genomic research? What about the right of the participants to withdraw from a study? The level of anonymity of genetic information in a database, the rights of participants and relatives to be informed of potential diseases, as well as many other important ethical issues related to genomic research will need to be addressed by regulations that are currently not in place or in use for ordinary research. The need for all the stakeholders to maintain the public trust cannot be underestimated. Any perceived breech of this trust will only serve to create an atmosphere of mistrust between participants and the research community, as well as with the regulatory agencies, and make it more difficult to conduct future research [25]. Some genetic information could violate the privacy and reputations of individuals or entire social groups, leading to discrimination. Safeguards will therefore be necessary to protect the welfare and privacy of participants of genomic research [33, 34].

Attempting to apply universal ethical principles to genomic research in a multicultural world with diverse healthcare systems and considerable variation in standards of healthcare presents yet another challenge. According to the International Ethical Guidelines for Biomedical Research Involving Human Subjects [35], research involving human subjects must not violate any universally applicable ethical standards, but acknowledge that, in superficial aspects, the application of the ethical principles, for instance, in relation to individual autonomy and informed consent, needs to take account of cultural values while fully respecting the ethical standards. Application of the universal principles of research ethics, albeit within a local context, will require well-trained individuals at several levels, including researchers and ethics committees, as well as regulatory agencies.

Genomic research is a specialised and very knowledge-intensive field [36]. Limitations in technical expertise and institutional capacity have constrained the progress of health research in Zambia. There is a clear need for a pool of technical expertise and improved funding for research and ethics monitoring if genomic research is to be effectively implemented. Members of RECs will require training on the intricacies of the legal and ethical implications of genomic research. Infrastructure will need to be provided for various levels of training in the relevant concepts of the applications of genomic research given the genetic literacy knowledge gap. Training members of IRBs/RECs, as well as interested researchers, will help to ensure informed ethical review processes for genomic studies. Public lectures, as well as information sessions, could also be used to educate the community and gauge the public perception of genomic research [37].

### Policy environment

The National Health Policy [38] provides for several measures with regards to health research, which include the provision of adequate funding for priority health research, linkage and integration between research, policy and action, construction of a national database and repository, as well as the development and implementation of an appropriate legal framework to guide research conduct. These measures are further highlighted in the National Health Research Policy [39].

Relevant policy frameworks will be required to guide the boundary within which genomic research can be applied in order to curtail scientific malpractice while ensuring the maximum benefits of health improvements. Additionally, it will be necessary to ensure that health research and associated outcomes primarily address the national interests.

The historical norm in Africa has been that the flow of samples and data has only occurred in one direction – out of Africa – creating a sense of mistrust and exploitation [40]. Genomic policy initiatives must, therefore, seek to change the notion that the problems faced by low- and middle-income countries such as Zambia can be solved by providing greater access to global knowledge resources and recognise the need for these countries to actively participate in knowledge generation.

Given the high costs associated with genomic research, it is imperative that the formulation of relevant policies entails public and/or external funding not only for the research but for infrastructure development as well. Local resource allocation to genomic research will be necessary as a demonstration of commitment to improve capacity for genomic research, in addition to initiatives from the NIH and Welcome trust [40, 41]. Local funding will also improve ownership and local interest.

Policies around genomic research will need to take into account the requirement for a publishing system that will drive the dissemination of research knowledge into locally accessible journals rather than high-cost international journals that effectively inhibit the ability of research to have meaningful impact on development. Open access publishing offers swift dissemination of research results, especially to places where these results can have meaningful impact. The existing conflict between open access dissemination of scientific discoveries and the protection of intellectual property rights, however, needs to be addressed if scientific discoveries are to be translated into national development, more so in the case of genomic research. A 2009 study on open access publishing [42] found that about 83% of the respondents supported the basic principle of open access and 90% indicated their willingness to publish in such journals. Approximately 68% of the respondents said that they would support the policies of research institutes, the government and donor agencies funding research to have publications from research activities deposited in open access institutional repositories.

Genomic research information also begs the question of privacy and confidentiality given that genomic information cannot be completely delinked from the individual [24]. Dissemination and access to genomic information, therefore, needs careful control in order to prevent identification of individuals or families. In a bid to formulate policies that address the issue, restrictive data release may be the way to go. This would regulate who has access to sensitive genomic information and what the information would be used for. Sound public policies will also need to be formulated in the area of both the provision and commercialisation of products of genomic research such as genomic tests. A 2003 review meeting by the Food and Drug Administration found that, although direct to consumer advertisements of genomic research products do tend to increase public awareness about diseases, they fail to accurately convey risk information [43]. Thus, the need for an appropriate legal and regulatory governance framework is imperative in order to address these and many other critical ethical issues [44].

## Legal framework

Looking back on the lessons learnt from research projects such as the Mazabuka Microbicide Research Project [45] and the passing of the National Health Research Act No. 2 of 2013 [46], it is clear that much needs to be done to prepare the legal landscape for genomic research in Zambia. The only piece of legislation that currently covers genomics is the Biosafety Act No. 10 of 2007 [47], which focuses mainly on genetically modified organisms or products, with a strong emphasis on agriculture. Zambia’s lack of directly pertinent genomic research regulation legislation, however, does not warrant concern, given the broad protection granted through the current National Health Research act [46]. The Act, in parts V and VI, provides a regulatory framework for health research involving humans or animals. It stipulates that: “*biological material for health research purposes may only be collected with the written consent of the individual,* […] *these materials may not be collected for any unspecified future health research activity or unspecified storage,* [and that] *Bio-banks shall be designated as such by the Minister*”. Part X of the act goes on to address issues regarding intellectual property rights. However, the Act does not make specific mention of genomics. The Policy, Monitoring and Research Centre conducted an analysis of the National Health Research Act and concluded that “*the Act had the potential to improve public health, labour productivity, create employment and promote economic growth*”. It went on further to highlight some of the challenges likely to be faced in the implementation of the act, namely the high cost of implementation and the creation of lengthy bureaucratic processes which may work against research advancement [48].

In order to provide a comprehensive legal mandate for genomic research, the existing pieces of legislation will require extensive review as has been done elsewhere in the United States, Europe, Asia, and Africa [49–53]. Massive changes, repeals and re-enactments of legislation pertaining to genomic research in Zambia will have to be undertaken creating an overhaul of the legal landscape; hence, the application of information from genomic research in health and non-health sectors is likely to cause a legal tsunami.

## Regulatory challenge

The National Health Research Act No. 2 of 2013 [46] makes provision for powers of the National Health Research Authority to, among others, withdraw the accreditation of a health researcher or research institution; ban health researchers and research institutions from carrying out research in Zambia; stop an ongoing health research activity; inspect any institution or site approved for the conduct of health research, including databases and bio banks; and to confiscate, impound and destroy, where necessary, biological materials obtained by any person in contravention of any provision of the Act.

Genomic research presents both national and international regulatory challenges. To think that such an area with great commercial, social, security and legal implications will be easy to regulate would be presumptuous. The various community engagement strategies will require monitoring so that effective ones can be adopted as best practices [54]. The regulatory challenge is based on the fact that, historically, the systems for regulation of research activities are weak with equally ineffective mechanisms to monitor research activities following ethics clearance. This makes it almost impossible for regulatory agencies in the country to guarantee the protection of the rights of research participants. This situation is aggravated by limited national legislation governing genomic research.

With the availability of genomic information comes the possibility of discrimination against individuals. Information about disorders tends to be of great interest to third parties such as insurance companies, law enforcement agencies or employers [55]. Regulation will be required for comprehensive protection of persons from genetic discrimination that may result from the abuse of sensitive genomic information. One example of regulation against genetic discrimination is the United State’s Genetic Information Non-discrimination Act of 2008 [56], which was passed into law to protect Americans against discrimination based on their genetic information when it comes to health insurance and employment. Additionally, the indiscriminate and uncontrolled use and exportation of human tissues and fluids has a bearing not only on human rights but poses a danger to biosafety and security of citizens. Thus, a multi-sectoral response to genomic research regulation may in the long run be more effective than a sectoral one.

Clear recommendations on how to deal with malpractices involving funders or researchers will be required. It is therefore evident that this is an area requiring not only ongoing consultations with all stakeholders, but allocation of financial and technological capacity to mount effective regulatory approaches.

## The opportunities at hand

The health improvement, cost efficiency of prevention of negative health consequences and the ease with which health conditions can be genetically detected before phenotypic expression is enormous. Genomic research has contributed to clinical advances in the study of rare diseases; the discovery of a single gene mutation in several instances has led to new therapeutic approaches to conditions that have perplexed clinicians for years [57]. Clinicians are now able to minimize the risk of prescribing the wrong dose by testing a patient’s genome for relevant variants in order to establish the appropriate dosage of medications, thereby making treatment more effective [58]. Furthermore, microbial genomics promises to improve the management of infectious diseases [59] and, with Zambia seeing a rise in the burden of non-communicable diseases such as cardiovascular diseases, diabetes, and cancers, genomics certainly offers a new frontier in healthcare.

The Zambia Health Research Act provides for the forging of health research collaborations while building local expertise by stipulating that, “*Health research shall not be conducted without the inclusion of a Zambian, who resides in Zambia, on the research team as a principal or co-principal researcher*” [46]. Opportunities for collaborative ventures, if well nurtured, can improve our understanding of health from a broader perspective. One such collaboration will see all African genomic projects sending a portion or all of their samples into one of several bio-banks. Guidelines and policies will be developed by the bio-bank working groups to govern sample movement [60].

On a secondary level, genomic research is an excellent platform for job creation, product development and innovation. Lessons can be drawn from the United States and India, where genomic research has significantly contributed to these countries’ economies – in the United States alone, Federal research investment has helped to generate nearly $1 trillion in economic impacts to date [61].

## Recommendations for action

In order for Zambia to fully benefit from the opportunity that genomic research presents, review or update of the current legal/policy framework will be necessary. There is an urgent need for the development of detailed guidance documents for the operationalisation of the policies. As has been already highlighted, any breach of trust, real or perceived, between the participants, community and regulators, can have a profound effect on the future of genomic research in Zambia. It is, therefore, imperative that trust is developed among stakeholders and that headway is made in demystifying genomic research for lay people.

Furthermore, if the challenges associated with the anticipated high costs of genomic research are to be overcome, adequate resources must be allocated to support ongoing scientific enquiry in this dynamic field. Training of local staff is one way to ensure that the benefits of genomic research to both the research community and public are optimised. The need to collaborate with relevant regional or international bodies towards creation of transparent and mutually beneficial information generation programs cannot be overstated. As it stands currently, legislation of research and ethics guidance is restrictive – developed as such to prevent exploitation and promote fairness. This has, however, had the unfortunate effect of limiting opportunities for African scientists to engage in international collaborations and use novel research methods. The solution lies in striking a balance between developing regulation that appropriately protects the interests of African researchers and research participants, and at the same time does not limit or restrict opportunities that could ultimately be beneficial for both.

## Conclusion

There is much room for improvement when it comes to the current research governance mechanisms, especially with regard to governing boards and research monitoring systems as well as reporting mechanisms and code of conduct adherence. Building capacity for genomic research will require input from multiple sectors in order to develop the appropriate regulatory frameworks (including the establishment of IRBs/RECs), building and maintaining physical infrastructure, and investing in human resources, equipment, and training as well as demand and supply for enhanced scientific research, based on a conviction that research, and particularly genomic research, can improve the lives of people and spur economic development.

## Abbreviations

IRB: Institutional review board
REC: Research ethics committee
